# Simulation, Fabrication and Microfiltration Using Dual Anodic Aluminum Oxide Membrane

**DOI:** 10.3390/membranes13100825

**Published:** 2023-10-08

**Authors:** Faheem Qasim, Muhammad Waseem Ashraf, Shahzadi Tayyaba, Muhammad Imran Tariq, Agustín L. Herrera-May

**Affiliations:** 1 Department of Electronics, Institute of Physics, GC University Lahore, Lahore 54000, Pakistan; 2 Department of Information Sciences, Division of Science and Technology, Township Campus, University of Education, Lahore 54000, Pakistan; shahzadi.tayyaba@ue.edu.pk; 3 Department of Computer Science, Superior University Lahore, Lahore 54000, Pakistan; imrantariqbutt@yahoo.com; 4 Micro and Nanotechnology Research Center, Universidad Veracruzana, Boca del Río 94294, Veracruz, Mexico; leherrera@uv.mx

**Keywords:** microfluidics, filtration, soft computing technique, anodization

## Abstract

Microfluidic devices have gained subsequent attention due to their controlled manipulation of fluid for various biomedical applications. These devices can be used to study the behavior of fluid under several micrometer ranges within the channel. The major applications are the filtration of fluid, blood filtration and bio-medical analysis. For the filtration of water, as well as other liquids, the micro-filtration based microfluidic devices are considered as potential candidates to fulfill the desired conditions and requirements. The micro pore membrane can be designed and fabricated in such a way that it maximizes the removal of impurities from fluid. The low-cost micro-filtration method has been reported to provide clean fluid for biomedical applications and other purposes. In the work, anodic-aluminum-oxide-based membranes have been fabricated with different pore sizes ranging from 70 to 500 nm. A soft computing technique like fuzzy logic has been used to estimate the filtration parameters. Then, the finite-element-based analysis system software has been used to study the fluid flow through the double membrane. Then, filtration is performed by using a dual membrane and the clogging of the membrane has been studied after different filtration cycles using characterization like a scanning electron microscope. The filtration has been done to purify the contaminated fluid which has impurities like bacteria and protozoans. The membranes have been tested after each cycle to verify the results. The decrease in permeance with respect to the increase in the velocity of the fluid and the permeate volume per unit clearly depicts the removal of containments from the fluid after four and eight cycles of filtration. The results clearly show that the filtration efficiency can be improved by increasing the number of cycles and adding a dual membrane in the micro-fluidic device. The results show the potential of dual anodic aluminum oxide membranes for the effective filtration of fluids for biomedical applications, thereby offering a promising solution to address current challenges.

## 1. Introduction

Microfluidic devices that have membranes with micro and nano pores are gaining significant importance in the fields of filtration, bio-medical applications and engineering [1,2,3,4,5,6]. The predictability of liquid and gas characteristics at the micro-level makes micro-fluidic devices an excellent alternative to conventional methods [7,8]. These devices handle very small amounts of fluids through channels with diameters in the range of a few micrometers. Microfluidic devices normally consist of a network of microchannels, chambers, valves, micro filters and micropumps. These are made of different materials, such as glass, silicon, polymers and metals [9,10]. These devices are fabricated for different applications. Different analyses are performed using these devices, including sample preparation, mixing, separation and analytic detection [11,12]. The commonly used techniques to fabricate microfluidic devices are injection molding, micromachining, soft lithography and hot embossing [13]. However, the method used for fabrication also depends on the applications for which the device is required. Various fabrication methods and steps can be used in combination to fabricate the microfluidic devices for specific applications. Microfluidic devices offer a wide range of applications in chemistry, biology, medicine, water purification and fluid filtration [14]. Microfluidic devices can be utilized for high-throughput drug screening, cell culture, DNA sequencing and point-of-care diagnostics. These devices and systems have advantages over standard laboratory procedures, such as reduced sample volume, increased sensitivity and enhanced accuracy [15].

Microfluidic membranes are a common type of microfluidic structures. A microfluidic membrane is a thin and porous structure used in a microfluidic device to separate two fluids or gases [16]. These membranes are designed with polymers, ceramics or metals, with pores ranging in size from a few nanometers to several micrometers [17]. Microfluidic membranes have a wide range of uses, including filtration, separation, hemodialysis and sensing [18,19]. These membranes are used to separate various kinds of cells in a biological sample or to filter out pollutants in a fluid. They are also used in biosensors to detect the presence of certain chemicals in a fluid. One advantage of employing microfluidic membranes is that they may be inserted directly into a microfluidic device. These devices can be used for proper control over fluid flow and component separation [14,20]. Furthermore, due to their high surface-area-to-volume ratio, the membranes can provide high sensitivity and rapid response time in sensing applications. In recent years, microfluidic devices have been fabricated using various novel materials. Different microfluidic devices have been developed by optimizing membrane characteristics and integrating membranes into devices to obtain improved sensitivity, selectivity and efficiency [21]. Membranes with various porosity topologies have been reported in the literature for their substantial influence on fluid purification and other applications [22]. Among the many materials, an anodic aluminum oxide membrane is categorized as an outstanding porous structure for micro-filtration applications such as bio-medical and fluid filtration [23,24]. The technique of visualizing the flow of microfluidic material and its penetration across the microfluidic membrane is considered as an important approach that needs substantial investigation. Aminullah et al. described the use of an Al-textured anodic aluminum oxide membrane in a microfluidic device with improved fluid permeability [25]. The contaminated fluid contains various types of bacteria, virus and unwanted substances. Therefore, an efficient fluid purification process is required. The pore size has remained important for the removal of bacteria (such as *Shigella*, *Escherichia coli*, *Vibrio* and *Salmonella*), viruses (such as Norwalk virus and rotaviruses),and protozoans (such as *Entamoeba*, *Giardia* and *Cryptosporidium*) in the fluid in the range of nanometers [26,27]. Because of the large difference in pore size, the removal of these containments in a single procedure is challenging. Therefore, filtration with multiple cycles and membranes can be useful to overcome such problems.

Various types of filtration methods have been used to filter out the impurities. The filters include reverse osmosis, mechanical filters, absorption filters, carbon filter, ion exchange, ceramic filter, activated alumina, sequestration filters and membranes. Membrane-based filters can be effectively used for microfiltration and ultra-filtration. Researchers have used various tools for the simulation of microfluidic devices for filtration modeling, prediction, permeation analysis and optimization. These simulation tools aid in predicting the process’s practical outcomes. Fuzzy and other expert systems, such as neural networks and machine learning techniques, have been used to analyze system behavior, while ANSYS and COMSOL were normally used to analyze the fluid flow in a microfluidic device [28,29,30,31].

The anodic aluminum oxide membrane has been reported as a part of device fabrication and as a template for the synthesis of nanostructures using different techniques and methods [32,33]. Different materials were used for the synthesis of nanoporous structures like metals, ceramics, polymers alumina, zirconia, titania and silica [34]. Among the different organic and inorganic materials, anodic aluminum oxide exhibits lots of useful properties and attractive attributes, like patterns and regular structures of pores, large surface area, controlled pores diameter, low cost of fabrication, impressive thermal conductivity and biocompatibility [35]. Due to such excellent properties, anodic alumina were reported for various applications. These applications include filtration [36], oxygen sensor [37], DNA sensor [38], biosensor [39], corrosion resistors [40], catalysts [41], cancer treatments [42], drug delivery [43], particle separation [44] and detectors [45]. Nanofiltration is an attractive method of ultra-filtration for the removal of pharmaceutically active compounds present in water [46]. Permeability for smaller compounds was investigated using various simulation-based methods [47]. Researchers also studied molecular dynamic simulation [48].

The novelty of the current work is to conduct the filtration by using two layers of anodic aluminum oxide membrane for the first time with smaller pore diameters from the range of no filtration to four and eight cycles of filtration. Overall, this work represents the simulation, the fabrication of an anodic aluminum oxide membrane and microfiltration for fluid purification. The system uses two different pore-sized membranes which provide the effective purification of fluid from different containments according to their size in the range of nanometers. The system is also designed to study the impact of the different process cycles required for filtration.

## 2. Methodology

### 2.1. Fuzzy Analysis

**Figure 1:**
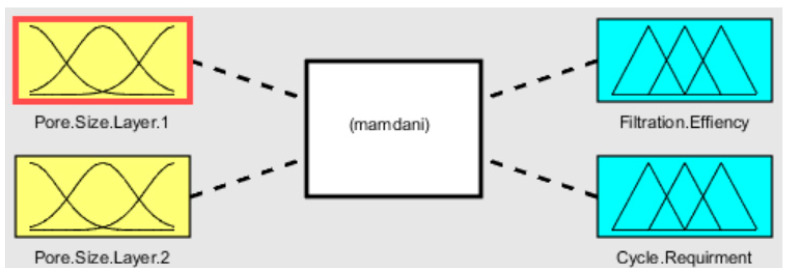
Fuzzy logic interface.

In this current research work, two anodic aluminum oxide membranes with different pore diameters have been designed and analyzed in a micro-fluidic device to filter the hazardous impurities from fluid. Fuzzy analysis predicts the output efficiency and the fluid purification cycle requirement based on the pore size of both layers. The fuzzy logic interface for the simulation is shown in Figure 1. Fuzzy logic is a soft computing technique and it works similar to human thinking. This technique is used for decision making based on multiple criteria. This technique is used to solve complex systems and problems in various fields. In particular, fuzzy-logic-based parametric estimation and optimization in the fields of biomedicine are useful. Here, fuzzy-based approach has been adopted for estimation of filtration efficiency and cyclic requirement of fluid to obtain purified fluid. The membrane with smaller pore size was taken as second layer with pore range of 70–120 nm. The membrane with larger pore size was installed as first layer with pore size in the range of 400–500 nm.

**Figure 2:**
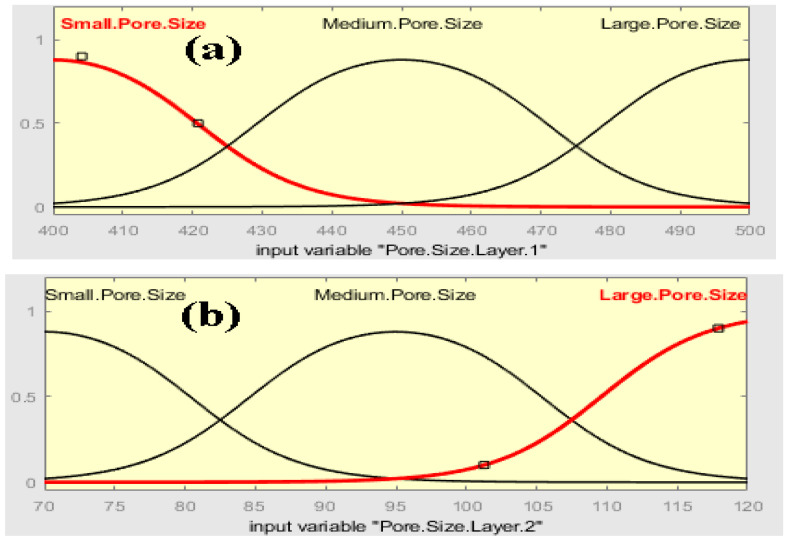
Membership function for input: (**a**) Pore Size Layer 1 and (**b**) Pore Size Layer 2.

The membership functions and ranges were adjusted in the membership function editor for fuzzy logic design algorithm. The membership functions for input are shown in Figure 2. The pore size for layer 1 and 2 is taken as small, medium and large with 400–500 nm and 70–120 nm ranges, respectively.

**Figure 3:**
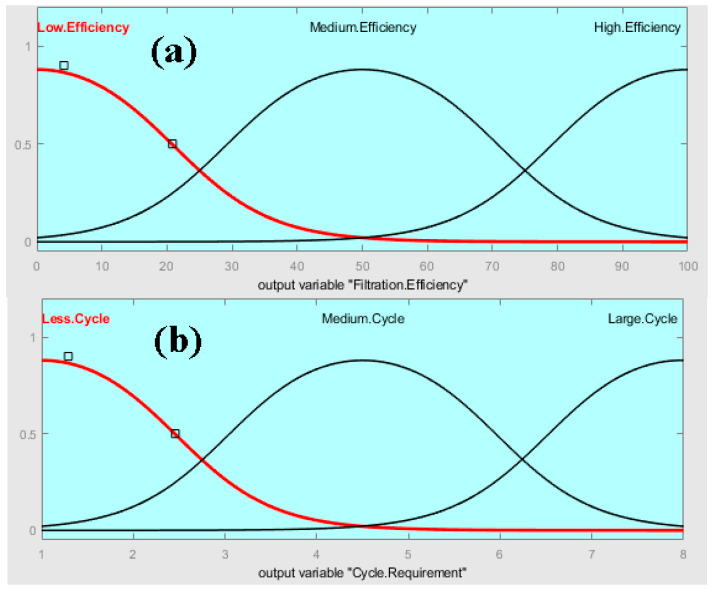
Membership function for output: (**a**) filtration efficiency and (**b**) cycle requirement.

The output membership function is shown in Figure 3. The membership function for output filtration efficiency is taken as low, medium and high efficiency with ranges from 0 to 100%. The membership function for output cycle requirement, as shown in Figure 3b, is taken as low, medium and large cycles ranging from 1 to 8 cycles, respectively. A total of nine rules were adjusted according to the Mamdani formula in the rule editor.

### 2.2. Microfluidic Simulation and Analysis

**Figure 4:**
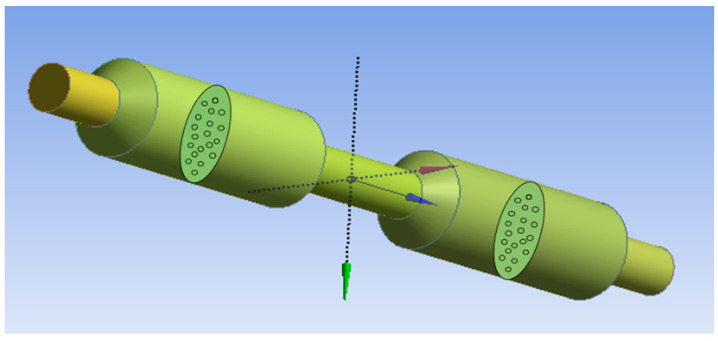
3D Filtration Model.

ANSYS fluent has been used for the 3D model of the filtration device, as shown in Figure 4. Two anodic aluminum oxide membranes were connected in series with each other. One end represents inlet and the other end acts as outlet of the filtration section. Newtonian fluid properties were taken into account for simulations through the microfluidic device. The pore size of the first anodic aluminum oxide membrane is taken as 400–500 nm and that of the second membrane is taken as 70–120 um, respectively.

**Figure 5:**
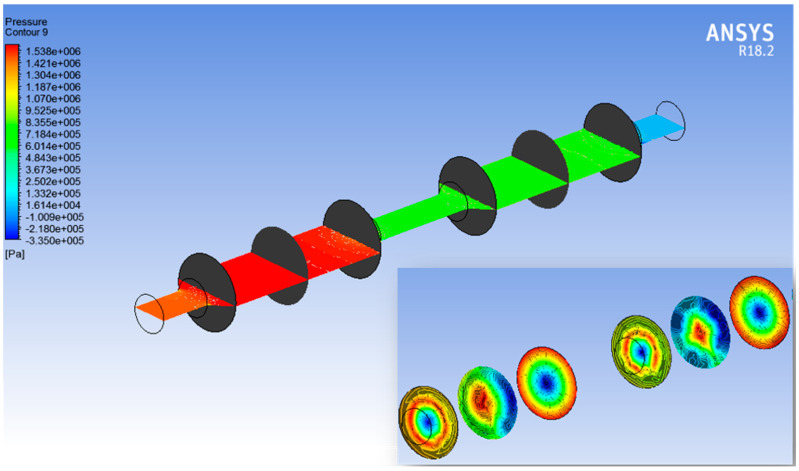
Global pressure distribution contour with local pressure distribution contour at bottom right corner.

**Figure 6:**
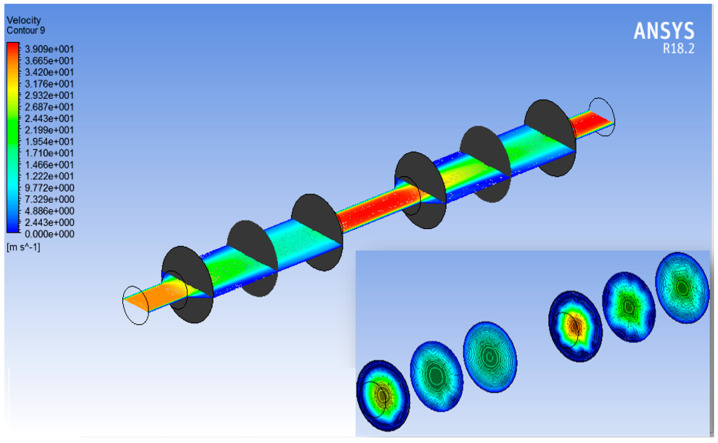
Global velocity distribution contour with local velocity distribution contour at bottom right corner.

The pressure and velocity variations inside the filtration setup were studied in the ANSYS fluent. The range in which the voltage and pressure were studied is −0.335 to 1.538 MPa for pressure, and the range of velocity is 0–39 cms^−1^. Boundary conditions were setup with maximum pressure at the inlet and zero pressure at the outlet. The global pressure and velocity variations inside the filtration setup are shown in Figure 5 and Figure 6, respectively, whereas the right corners of both Figure 5 and Figure 6 represent local contours for pressure and velocity distribution. Six vertical and one horizontal planes in the setup are given for better understanding of the pressure and velocity distribution.

The flow rate of the membrane depends on the radius, the shape of membrane pores and the membrane size, as given by Poiseuille’s law in Equation (1):

(1) $$Q=\frac{\pi \;D_{i}^{4}(\nabla P)}{128\mu (L)}$$

Here, *Q* denotes fluid flow rate, *D_i_* denotes internal diameter, ∇*P* denotes the pressure variation, *μ* denotes fluid viscosity and *L* is the channel length.

The rate of filtration can be calculated by using Darcy’s law in Equation (2):

(2) $$\frac{dV}{dt}=\frac{KA}{u}\frac{P}{l}$$

where *V* is filtrate fluid volume, *K* is permeability coefficient, *A* is area of membrane filter, u is fluid viscosity, *P* is pressure gradient and *l* is membrane thickness.

### 2.3. Fabrication of Anodic Aluminum Oxide Membrane

#### 2.3.1. Materials

Aluminum sheets, de-ionized water, isopropyl alcohol, phosphoric acid (H_3_PO_4_), chromic acid (H_2_CrO_4_) and oxalic acid (C_2_H_2_O_4_) have been used in the experiment. All the raw material was purchased with 99.98% purity. The aluminum substrate was rolled aluminum that had a 10 um thick, 99.98% pure electroplated aluminum layer on top. Before anodization, the samples were divided into 35 mm × 50 mm pieces and cleaned. Two aluminum sheets were taken after complete cleaning using de-ionized water and isopropyl alcohol (IPA) followed by electrochemical polishing and cleaning with ethanol, which were performed to obtain a smooth surface of the substrate. The two sheets were then subjected to anodization.

#### 2.3.2. Methods

The anodic aluminum oxide membrane was fabricated using the same methodology as reported in our previous work with little modification of the parameters [19]. The membrane was fabricated using two-step anodization, including mild and hard anodization. The first step of anodization, known as mild anodization, included 0.3 M oxalic acid as an electrolyte solution with a voltage ranging from 110 to 150 V for 5 min (the variable voltage is studied for the generation of pores with small and large pore size, respectively, and etched for pore widening). The prepared membrane after mild anodization was etched using a mixture of 4% poshporic acid and oxalic acid for 30 min. After etching the two layers of aluminum, it was passed through the hard anodization, which is similar to mild anodization but the time of anodization is much longer than the mild anodization. In the second step of anodization (Hard Anodization), the same oxalic acid was used as an electrolyte, however, the voltage was set to 110 V and 150 V for 2 h and etched to remove any impurities and the barrier layers. The etching process for the hard anodization was similar to the mild anodization.

#### 2.3.3. Characterization

Both anodized membranes were then studied using a scanning electron microscope (SEM) before filtration and after filtration to check for the proper removal of the impurities and the surface structure. The morphology was imaged by using a scanning electron microscope, Model: Vega3, Tuscan.

### 2.4. Filtration Setup

**Figure 7:**
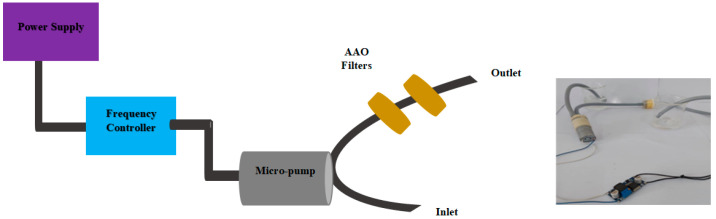
Schematic and actual setup of filtration.

The micro-fluidic system consists of two anodic aluminum oxide membrane filters fabricated using mild and hard step anodization. The pore size of the membrane in first filter is kept greater in comparison to the second filter in order to enhance filtration efficiency. The pore size is set in such a way that the particles, including bacteria and other hazardous materials, are completely removed. The first filter blocks unwanted material of a larger size from the contaminated fluid, while the second filter blocks the smaller impurities present in the fluid which are required to be removed in order to generate a purified and clean fluid. An MP6-micropump and frequency controller are connected to manipulate and rectify the fluid flow. The schematic and actual setup of the filtration used for fludic filtration with two membranes are shown in Figure 7.

## 3. Results and Discussion

### 3.1. Fuzzy Analysis Results

**Figure 8:**
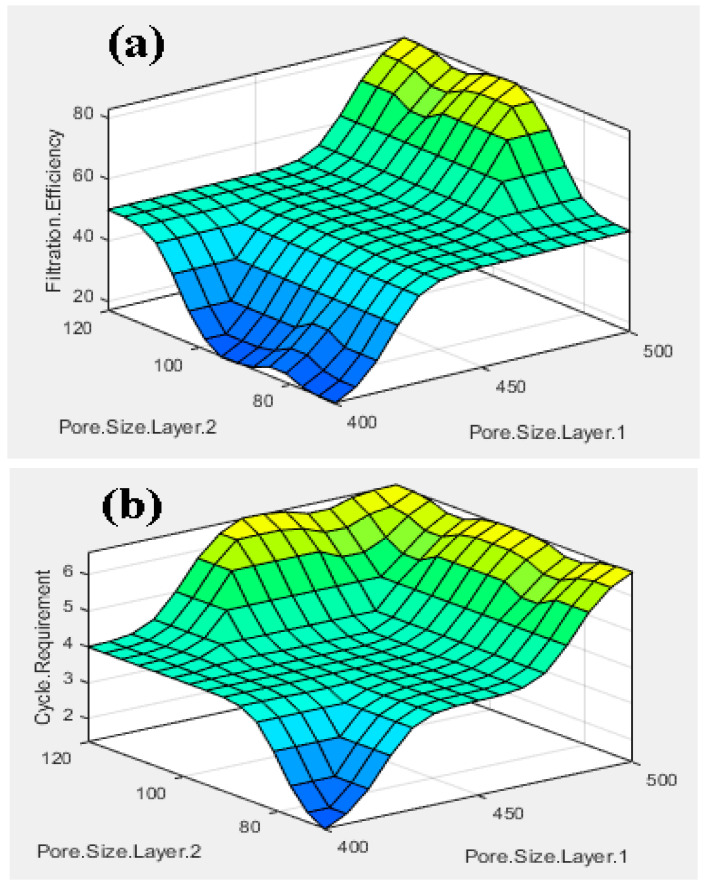
3D graphs between input Pore Size Layer 1 and Pore Size Layer 2 with outputs: (**a**) filtration efficiency and (**b**) cycle requirement.

The fuzzy-rules-based three-dimensional (3D) output graphs based on the input are shown in Figure 8. Figure 8a shows the graph between the input pore size layer 1 and pore size layer 2 with the filtration efficiency as output. Larger pore size of the layer will be helpful for the higher removal of unwanted particle sizes. The larger unwanted particles will be filtrated out, creating a blockage on the pores of the membrane. In the next cycle, hazardous contaminates with smaller size will be filtrated because of the closing of the pores of the membrane, resulting in better filtration. Figure 8b shows the 3D graph between pore size layer 1 and pore size layer 2, with the cycle requirement as output. With larger pore size, more cycles can be taken to perform a better filtration and improve the filtration efficiency. Based on the points extracted from the fuzzy logic controller, the filtration efficiency and cycle requirements can be studied.

**Figure 9:**
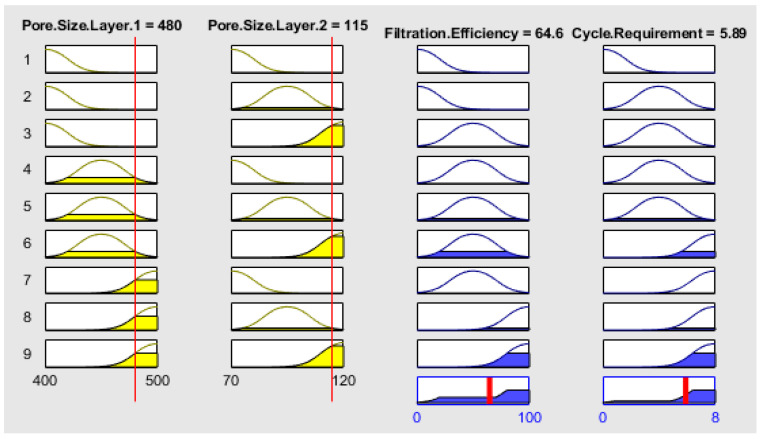
Rule viewer of the dual anodic aluminum oxide membrane for fluid filtration.

Figure 9 shows the rule viewer, which provides the real-time estimated value of the filtration efficiency and the cycle requirement. The simulated values from the work are compared with the calculated values using the Mamdani model.

Based on the values of the rule viewer, the filtration efficiency and cycle requirements were calculated by using the Mamdani model. Then, the simulated and calculated values were compared. Table 1 shows the comparison between the simulated and calculated values for the outputs (filtration efficiency and cycle requirement). The error between the values is significantly less than 1, which shows that the inputs and their impact on the outputs are as per the logical and desired results.

### 3.2. ANSYS Fluent Results

**Figure 10:**
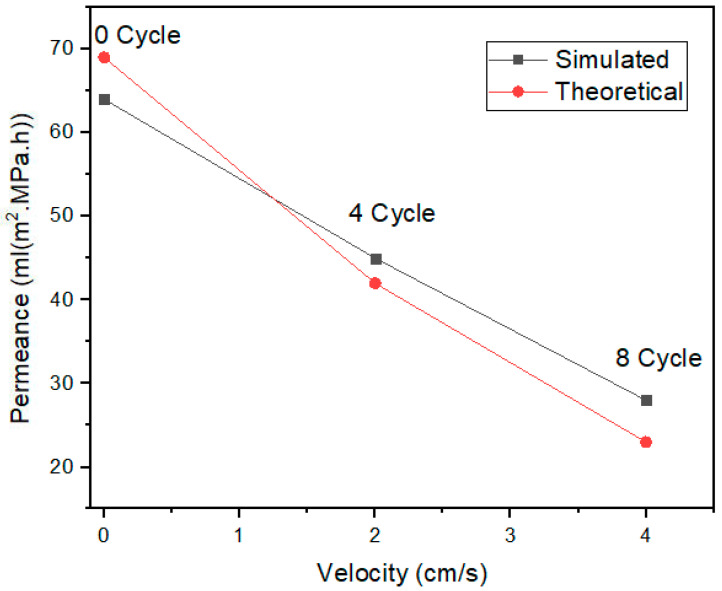
Simulated and theoretical results for velocity impact for 0, 4 and 8 Cycles.

From the ANSYS fluent results, the simulated and theoretical results for the velocity profile are shown in Figure 10. The fluid flows when the pressure is applied on one end of the membrane. The flow rate in the first cycle is higher in both the anodic aluminum oxide membranes, as shown in Figure 10.

With the increase in the number of cycles for the fluid flow, the flow rate through the membrane decreases. This is mainly attributed to the deposition of the impurity materials from the fluid, including bacteria and other particles, on the membrane which clog the membrane. Even if the pressure is higher, due to clogs it become difficult for the fluid to pass through the pores, resulting in a decrease in the velocity. The permeance decreases from 70 mL (m^2^·MPa·h) to 25 mL (m^2^·MPa·h) when the filtration cycle changes from 0 to 8. The membrane does not degrade when pressure is applied on it, mainly due to the behavior of the fluid, if the fluid will behave in neither an acidic nor basic manner.

### 3.3. AAO Template Morphology Results

**Figure 11:**
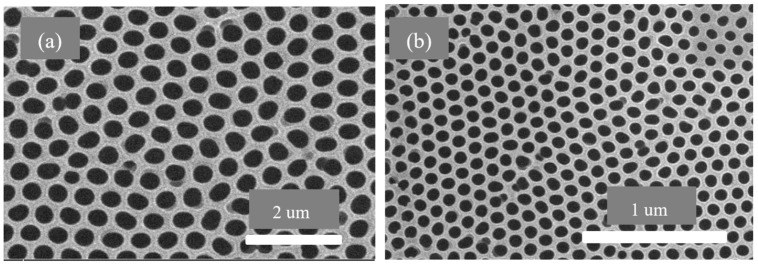
SEM graphs of anodic aluminum oxide membrane before using in filtration: (**a**) first anodic aluminum oxide membrane with pore size 400–500 nm and (**b**) second anodic aluminum oxide membrane with pore size 70–120 nm.

The SEM graph of the pre-filtration membranes is shown in Figure 11. Figure 11a shows the anodic aluminum oxide membrane with larger pore size ranging from 400 to 500 nm for the filtration of large molecules from the fluid sample. Figure 11b shows the second anodic aluminum oxide membrane with smaller pore size ranging from 70 to 120 nm, which acts as another layer to remove smaller impurities from the fluid sample under consideration.

**Figure 12:**
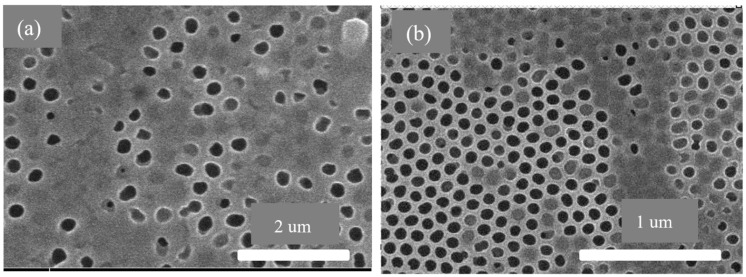
SEM graphs of anodic aluminum oxide membrane after 4 cycles of filtration: (**a**) first anodic aluminum oxide membrane with pore size 400–500 nm and (**b**) second anodic aluminum oxide membrane with pore size 70–120 nm.

Figure 12 shows the SEM graphs after four cycles of filtration. It clearly shows that clogs are present on the pores which are the materials in the samples. Those materials were required to be removed from the contaminated fluid during filtration. Figure 12a shows the first layer of the anodic aluminum oxide membrane blocked due to contaminates in the samples. Figure 12b shows the second layer of the anodic aluminum oxide membrane pores blocked due to smaller hazardous and contaminant materials.

**Figure 13:**
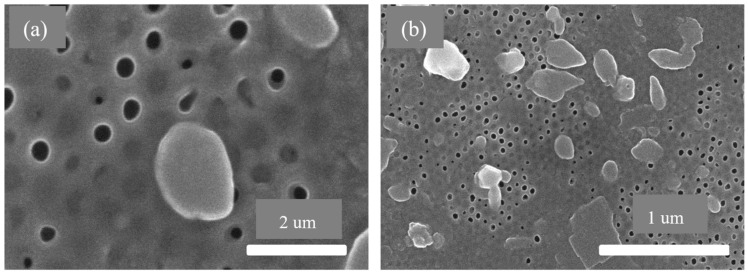
SEM graphs of anodic aluminum oxide membrane after 8 cycles of blood filtration: (**a**) first anodic aluminum oxide membrane with pore size 400–500 nm and (**b**) second anodic aluminum oxide membrane with pore size 70–120 nm.

Figure 13 shows the SEM graphs after eight cycles of filtration. The number of clogs has been increased now and the fluid flow rate has been decreased, due to the fact that the fluid flow cannot be easily possible because of the pore clogging. Figure 13a shows the first layer of the anodic aluminum oxide membrane blocked due to the storage of unwanted material like protozoans in the pores of the membrane. Figure 13b shows the second layer of the anodic aluminum oxide membrane blocked due to impurities present in the samples.

### 3.4. Filtration Analysis

**Figure 14:**
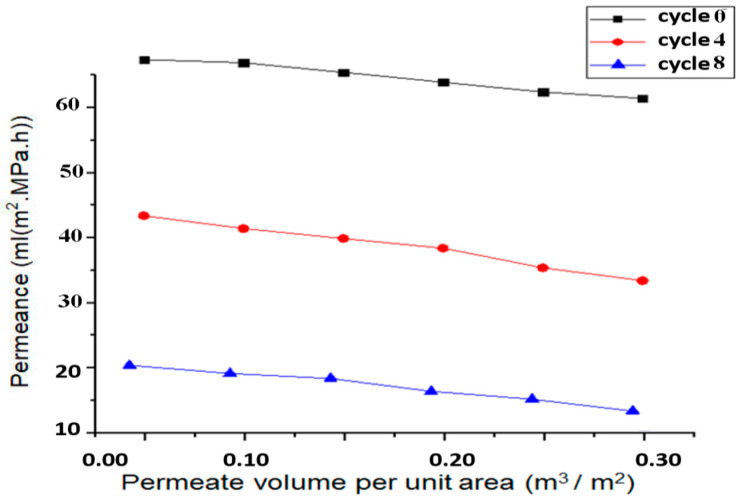
Graph between permeance and permeate volume per unit area.

The permeance and the permeate volume per unit area is studied for the analysis of the fluid filtration using the prepared membrane. It is clear that the permeance decreases with the decrease in the cycle, as shown in Figure 14.

The decrease in the permeance is mainly due to the fact that the flow rate decreases per unit area due to the clogging of the pores of the anodic aluminum oxide membrane. The simulated and calculated results are closely related to the experimental results. The decrease in permeance with the permeate volume per unit area sees a decline of approximately 5 mL (m^2^·MPa·h) when the permeate volume per unit area changes from 0 to 0.3 m^3^/m^2^ for all the respective filtration cycles.

On the basis of the two-layer anodic aluminum oxide setup, purification and filtration can be easily carried out based on different types and size of impurities (bacteria) in the samples. Those virus and protozoa can be removed which are in the range of the reported pore size of the filter. This method provides a better approach to improving filtration with better efficiency and more cycles for filtration to generate an impurity-free fluid.

Table 2 shows the comparative analysis between the literature and the current work for anodic aluminum oxide membranes with respect to their application and other parameters. The table shows that different morphological structures with an anodic aluminum oxide template have been reported in the literature, with a pore size range of 30–500 nm for the filtration of fluids and solids. However, the reported device in this work has the novelty of having two layers of anodic aluminum oxide membrane with different pore size and better fluid flow through the microfluidic device.

The current study has been limited to investigating the clogging of filters with filtration for various cycles of contaminated fluids. In future work, the comparison between the fluid contaminants size, the pore size of filter and the properties of the fluid will be presented before and after the filtration of each cycle. This study would provide a useful pathway for researchers working in microfluidic devices and filtration.

## 4. Conclusions

This work has been carried out using dual anodic aluminum oxide membranes for fluidic filtration. The conclusions drawn from the presented work include the following findings:

1. The fuzzy-rule-based 3D graphs establish connections between the input pore size in Layer 1 and Layer 2, the filtration efficiency and the cycle requirements as outputs. The larger pore size in Layer 1 was found to enhance the removal of unwanted particles, resulting in pore blockage and the subsequent filtration of smaller contaminants, thereby improving filtration efficiency. The dual membranes were analyzed using soft computing techniques. A fuzzy analysis shows that the membrane pore size is a factor that greatly impacts the filtration efficiency and number of cycles required for purification. With larger pore size, more cycles can be taken for filtration, resulting in the better efficiency of the filtration process.
2. The ANSYS simulation results shows that the fluidic flow reduces with an increase in the number of cycles, mainly due to the clogged pores due the impurities present in the samples. The permeance decreased as the filtration cycles progressed, primarily due to impurity-induced pore blockage.
3. The SEM results of the fabricated AAO membrane show the morphology of AAO membranes before filtration, featuring two layers with different pore sizes (400–500 nm and 70–120 nm). SEM images after four and eight filtration cycles demonstrated increased pore clogs and decreased flow rates, which is attributed to the accumulation of contaminants within the pores.
4. Finally, the results show that the overall filtration efficiency can be improved using the dual AAO membranes in comparison to using single membrane. The number of cycles has been increased from four to eight, in comparison to from four to six as reported in the literature. This AAO low-cost membrane can be used effectively for fluid filtration in biomedical applications. The higher number of cycles for filtration gives more purified fluid.

## Figures and Tables

**Table 1:** Difference between the simulated and the calculated values.

Quantities	Filtration Efficiency (%)	Cycle Requirement (Number)
Simulated Value	64.6	5.89
Calculated Value	64.65	5.88
Error	0.05	0.01

**Table 2:** Comparison of current study results with the literature.

Reference	Membrane Type	Number of Layers for the Membrane	Pore Size (nm)	Fluid Flow Velocity/Flux	Filtration Application
Aminullah 2018 [25]	Al-textured AAO membrane	Single	31.25	Fluid flow velocity dependent on the viscosity of the fluid	Flow of fluid, permeability of acetone, ethanol, dimethylformamide, methanol, cyclohexane, isopropyl alcohol, water and n-butanol
Jooyoung 2011 [49]	Polyrhodanine-modified anodic aluminum oxide membrane	Single	150	-	Removal of heavy metal ions from wastewater
Chein 2018 [50]	Tubular AAO films	Single	60	-	Drug delivery, liquid filters, gas filters and energy applications
Yatinkumar 2020 [24]	Nanoporous AAO Membrane	Single	50–90	-	Nano-filtration
Huang 2020 [51]	CO_2_-gated AAO-based nanocomposite membrane	Single	210–260	Flux—50–500 L m^−2^ h^−1^	De-emulsification
Phuong 2016 [52]	Functionalized nanoparticles embedded in anodic aluminum oxide templates	Single	150	Flux—48.19 g/sm^2^	Sand filtration
Manzoor [19]	Tunable AAO membrane	Single	50–100	Fluid flow velocity 0–3 cm/s	Microfludic filtration for biomedical application
Presented work	Dual-layer AAO membrane	Double	70–500	Fluid flow velocity 0–4 cm/s	Contaminated fluid purification for biomedical application

## Data Availability

Most of the steps and details have been provided in the manuscript. However, more detail and information can be obtained from the authors.
